# Novel Bovine Papillomavirus Type Discovered by Rolling-Circle Amplification Coupled with Next-Generation Sequencing

**DOI:** 10.1371/journal.pone.0162345

**Published:** 2016-09-08

**Authors:** Flavio R. C. da Silva, Samuel P. Cibulski, Cíntia Daudt, Matheus N. Weber, Lorena L. B. Guimarães, André F. Streck, Fabiana Q. Mayer, Paulo M. Roehe, Cláudio W. Canal

**Affiliations:** 1 Laboratório de Virologia – Faculdade de Veterinária, Universidade Federal do Rio Grande do Sul, Porto Alegre, Rio Grande do Sul, Brazil; 2 Centro de Ciências Biológicas e da Natureza, Universidade Federal do Acre Rio Branco, Acre, Brazil; 3 Universidade de Caxias do Sul, Caxias do Sul, Rio Grande do Sul, Brazil; 4 Laboratório de Biologia Molecular – Instituto de Pesquisas Veterinárias Desidério Finamor (IPVDF), Fundação Estadual de Pesquisa Agropecuária, Eldorado do Sul, Eldorado do Sul, Rio Grande do Sul, Brazil; 5 Departamento de Microbiologia Imunologia e Parasitologia – Laboratório de Virologia, Universidade Federal do Rio Grande do Sul, Porto Alegre, Rio Grande do Sul, Brazil; Universidad de Chile, CHILE

## Abstract

Currently, fifteen bovine papillomavirus (BPV) types have been identified and classified into four genera: *Deltapapillomavirus*, *Epsilonpapillomavirus*, *Dyoxipapillomavirus*, and *Xipapillomavirus*. Here, the complete genome sequence of a new BPV type (BPV 04AC14) recovered from a papillomatous lesion is reported. The genome is 7,282 bp in length and exhibits the classic genetic organization and motifs of the members of *Papillomaviridae*. Maximum likelihood phylogenetic analyses revealed that BPV 04AC14 clusters with members of the *Xipapillomavirus* genus. The nucleotide sequence of the L1 capsid protein of the novel BPV is closely related to its counterpart, BPV3, with which it shares 79% similarity. These findings suggest that this virus is a new BPV type of the *Xipapillomavirus* genus.

## Introduction

Papillomaviruses (PVs) are small viruses whose genomes consist of double-stranded DNA molecules of approximately 8 kb; PVs are widely distributed and probably infect all amniotes [1]. Most PVs are part of the skin microbiota; however, in some cases, infections by certain types manifest in distinct clinical presentations, from highly productive, self-limited warts to invasive cancers [2]. In cattle, bovine papillomavirus (BPV) infections are probably primarily asymptomatic, although on occasion, certain BPV types can induce skin warts or neoplasias in the mucosa of the urinary bladder and upper digestive tract [3,4].

PVs are classified in the *Papillomaviridae* family and subdivided into 39 genera and several species, types, subtypes and variants. This discrimination is based on the degree of nucleotide sequence diversity of the L1 gene [5–7]. Currently, fifteen BPVs have been reported, in contrast to the >200 types of viruses identified in humans (HPVs) (http://pave.niaid.nih.gov). The BPVs are assigned into four genera: the genus *Deltapapillomavirus*, with one species; *Deltapapillomavirus 4*, comprising four types (BPV1, 2, 13 and 14); the genus *Epsilonpapillomavirus*, comprising the species *Epsilonapapillomavirus 1*, with two types (BPV5 and BPV8); *Dyoxipapillomavirus*, which comprises *Dyoxipapillomavirus 1* species (BPV7); and the *Xipapillomavirus* genus, composed by the *Xipapillomavirus 1* (BPV3, 4, 6, 9, 10, 11 and 15) and *2* species (BPV12) (http://pave.niaid.nih.gov).

The multiply primed rolling-circle amplification (RCA) strategy has been successfully used for the identification of the circular genomes of a number of viruses [8], e.g., anelloviruses [9], circoviruses [10] and papillomaviruses [11]. The method utilizes bacteriophage ϕ29 DNA polymerase for the selective amplification of circular DNA [8]. Unlike PCR, the primers used in the amplification reaction are exo-resistant random. Therefore, as the technique does not need any specific primer, previous knowledge of the nucleotide sequences is not necessary. Furthermore, ϕ29 DNA polymerase has linear kinetics at 30°C, eliminating the need for thermal cycling. By strand displacement synthesis, repeated copies of the complete genome are synthesized, leading to a high molecular mass double-stranded DNA.

In 2014, a specimen consisting of papillomatous-like lesions was received in the laboratory for the production of a BPV autogenous vaccine. Confirmation of the presumptive diagnosis and typing of the BPV involved was performed by PCR with consensus PV primers and Sanger sequencing [12]. Nucleotide sequencing indicated a BPV type not previously described. The full genome of this novel BPV type was recovered directly from the papillomatous lesions by multiply primed rolling-circle amplification (RCA) followed by NGS. The genome sequence was characterized and the phylogenetic relationship between this novel BPV and the other BPVs was determined.

## Materials and Methods

### Sample

A specimen consisting of ~20 grams of papillomatous lesions (skin warts; sample 04AC14) was received in the lab. The specimens were derived from one animal in Acre State (within the Brazilian Amazon region), clinically diagnosed as papillomatosis, and collected for the production of a BPV autogenous vaccine. The lesions were removed using scalpels after local anaesthesia (performed with 2% lidocaine). The sample was individually wrapped and stored at −20°C for DNA extraction and in 10% buffered formaldehyde for histopathological analyses. DNA extraction, PV consensus PCR and Sanger sequencing was present in S1 File.

### Histopathology

Tissue were fixed in 10% buffered formalin, trimmed, and processed routinely for histopathology. Tissue sections were cut at 3 μm and stained with haematoxylin and eosin (HE).

### Rolling-circle amplification (RCA)

Multiply primed rolling-circle amplification (RCA) was performed as previously described [10,13]. Briefly, 100 ng of total DNA (2 μL of 50 ng/μL solution) from papillomatous tissue was denatured at 95°C for 5 minutes and immediately cooled on ice. Twenty-three microlitres of a previously prepared solution containing 1.5 mM of each dNTP (Invitrogen), 6.2 mM random exonuclease-resistant hexanucleotides (Thermo), 2 U of ϕ29 DNA polymerase (Thermo) and 2.5 μL of reaction buffer [50 mM Tris/HCl pH 7.5, 10 mM MgCl_2_, 10 mM (NH_4_)_2_SO_4_, 4 mM dithiothreitol] were added to denatured DNA. The amplification solution was incubated for 18 hours at 30°C, followed by 10 min at 65°C to inactivate the enzyme. The amplicon was electrophoresed in a 0.8% agarose gel and visualized on a UV light source after ethidium bromide staining. The RCA products were purified with a commercial kit (GFX^™^ Purification Kit; Amersham Biosciences).

### High-throughput sequencing and sequence analysis

RCA DNA was tagged and fragmented using the Nextera DNA Library Prep Kit (Illumina) according to the manufacturer’s instructions for the preparation of DNA libraries. After amplification via a limited-cycle PCR program, PCR cleanup was performed with Agencourt AMPure XP beads (Beckman Coulter). The library was sequenced in a MiSeq System (Illumina) using a MiSeq Reagent Kit V2 (2x150 cycles).

The data were *de novo* assembled using SPAdes genome assembler (version 3.6) [14]. Open reading frame (ORF) predictions and genome annotations of the 04AC14 genome were performed with the aid of Geneious software (version 8.1.4). Gene and protein comparisons were performed in the programmes BLASTn and BLASTp. Sequence of the BPV 04AC14 was deposited in GenBank under accession number KX098515.

### Phylogenetic inferences

Local sequence alignments were constructed to determine the sequence identity with BLASTn [15]. Representative PV sequences were retrieved from GenBank. Nucleotide alignments were performed using MUSCLE software [16].

The best selection model to generate the phylogenetic trees was selected with the programme Modeltest 3.7 [17]. A phylogenetic tree with 1000 bootstrap resamples of the alignment data sets was generated using the Maximum Likelihood method in MEGA5 [18]. Bootstrap values (based on 1000 replicates) for each node are given.

## Results and Discussion

### *De novo* sequencing and genome assembly

Illumina MiSeq (2×150 cycles run) generated a total of 110,820 high quality paired-end reads. These sequences were *de novo* assembled into 3,885 contigs using SPAdes 3.6 assembler. These contigs were analysed using BLASTn/BLASTx with the National Center for Biotechnology Information (NCBI) databases. One contig (named Node_1) related to *Papillomaviridae* with a circular genome of 7,282 nucleotides (nt) was identified. This contig was composed by 857 reads (mean coverage ~17). The remaining contigs were either related to the bovine genome or to unknown sources.

### Genomic characterization of a new BPV type

**Fig 1:**
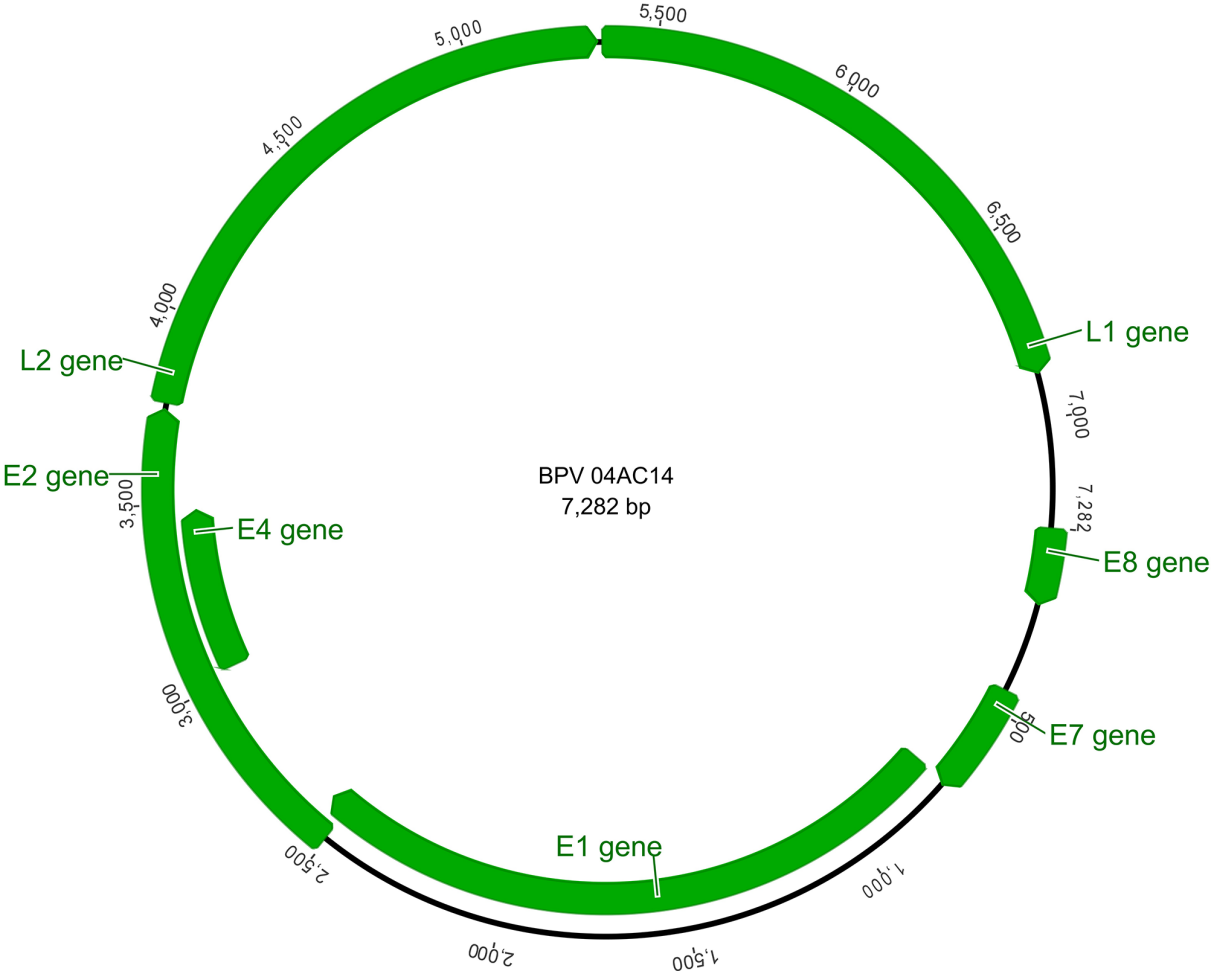
Genomic organization of BPV 04AC14. The first position of the BPV 04AC14 genome was set as the first ATG of the E8 ORF. The ORFs were identified with the aid of the ORF Finder (NCBI). The figure was drawn in Geneious 8.1.4.

The PV recovered genome is 7,282 bp in length, arranged in a circular DNA molecule with a GC content of 42.5%. Seven BPV ORFs were identified in the positive strand: five of those corresponded to the genes coding for early proteins (*E8*, *E7*, *E1*, *E2* and *E4*) and two genes (*L1* and *L2*) coded for the late capsid proteins. A long control region (LCR) of 399 bp was recognized between the *L1* and *E8* gene, located at nt positions 6,884–7,282. The major genome features of 04AC14 BPV are shown in Fig 1.

Whole-genome sequence alignments revealed that the closest related PVs were BPV3 (AF486184; 77% of identity to 04AC12), BPV6 (AJ620208; 74%) and BPV4 (X05817; 73%). When each ORF was compared with other PVs, the degree of nucleotide identity varied between 74% and 81% (Table 1).

**Table 1:** Main features of the BPV 04AC14 genome.

	Position	Size (nt/aa)	Predicted MW (kDa)	Best blastn hit/Identity
**El**	726–2,564	1,839/612	69.6	BPV3 (AF486184)/81%
**E2**	2,506–3,753	1,248/415	46.8	BPV3 (AF486184)/77%
**E4**	3,029–3,487	459/152	17.7	BPV3 (AF486184)/76%
**E7**	440–736	297/98	10.8	BPV3 (AF486184)/80%
**E8**	1–204	204/67	7.7	BPV11 (AB543507)/77%
**L1**	5,354–6,883	1,530/509	57.6	BPV3 (AF486184)/79%
**L2**	3,765–5,342	1,572/525	56.8	BPV3 (AF486184)/75%
**LCR**	6,884–7,282	399/-	-	BPV3 (AF486184)/74%

**Fig 2:**
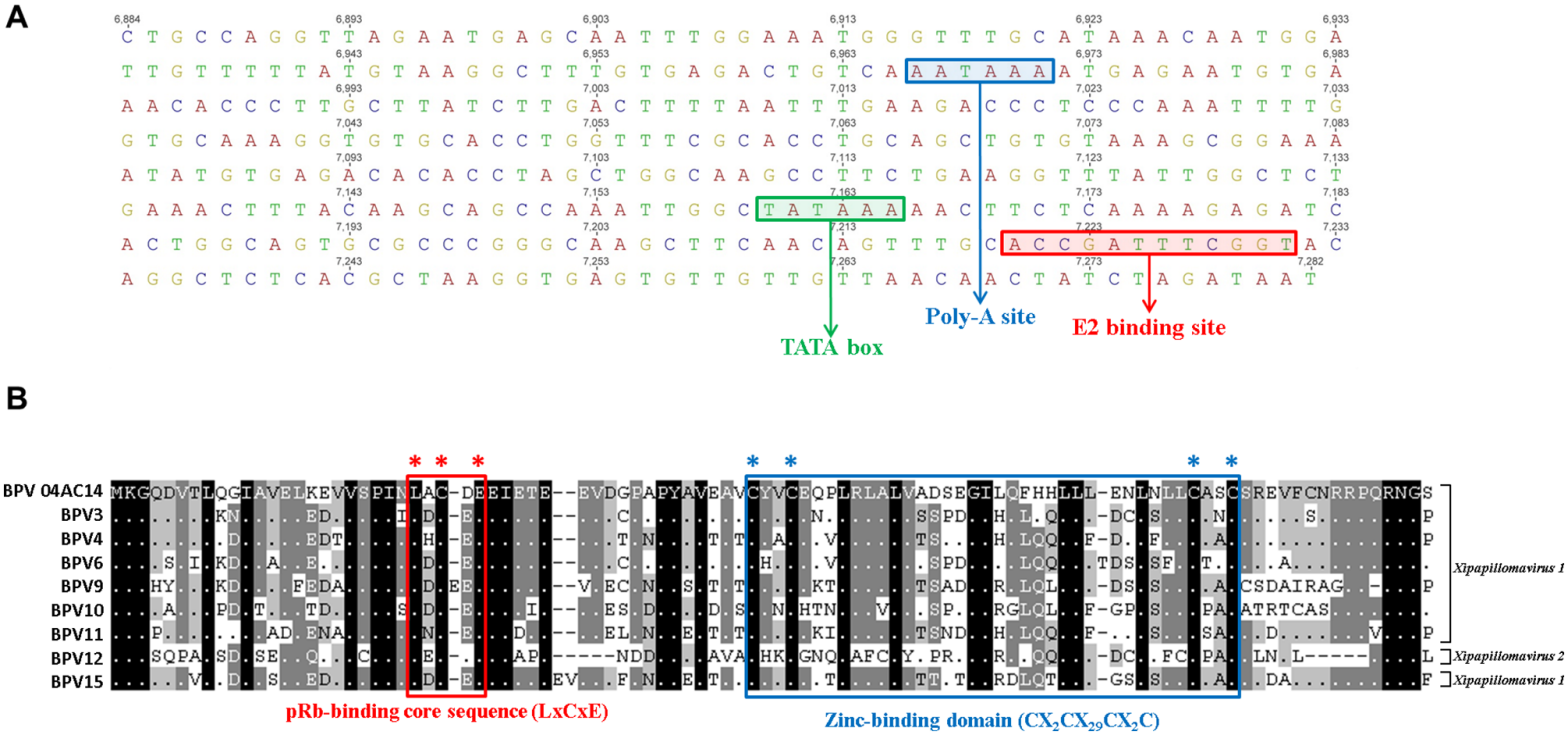
Features of the BPV 04AC14 non-coding region and E7 protein. (A) Non-coding region: coloured boxes display the genomic locations of the E2 binding site (ACCN_5_GGT), polyadenylation site (AATAAA), and TATA box (TATAAA). (B) Amino acid alignment of E7 proteins with corresponding proteins of closely related types from *Xipapillomavirus 1* and *2*. The location of the pRb-binding core sequence (LXCXE) is marked with a red box, and the location of the zinc-binding domain (CX_2_CX_29_CX_2_C) is indicated by a blue box. Multiple sequence alignments were performed in MUSCLE, and the figure was drawn in GeneDoc.

The LCR of BPV 04AC14 contained typical PV features [19] that hold regulatory elements for virus replication and control the transcription of transforming genes. The LCRs of mucosal epitheliotropic PVs possess a similar genome organization, which typically includes a promoter region, an enhancer region and a highly conserved distribution of E2 DNA binding sites [20]. BPV 04AC14 lacks a second LCR, similar to the majority of BPVs. Both E1 and E2 bind to the origin of virus replication, located in the LCR, and activate genomic DNA replication. Most PVs possess one E1-binding site (E1BS) and at least two E2-binding sites (E2BS) [21]. Nevertheless, the BPV 04AC14 LCR shows only one copy of E2BS (ACCN_6_GGT). In addition, the BPV 04AC14 LCR possesses one poly-A site (AATAAA) and one TATA box (TATAAA), both of which are important transcription and replication regulatory elements. The E2BS, poly-A site, and TATA box are located at positions 7,220–7,231, 6,966–6,971 and 7,160–7,165, respectively (Fig 2A).

The early region of PV genomes encodes the non-structural viral proteins involved in viral DNA replication, transcription and cell transformation [22]. The early region of BPV 04AC14 encodes 5 ORFs: *E8* (204 bp), *E7* (297 bp), *E1* (1,839 bp), *E2* (1,248 bp) and *E4* (459 bp).

The BPV 04AC14 genome encompasses a putative *E8* gene. This gene encodes a protein that is chemically and functionally similar to the protein encoded by the HPV *E5* gene and may also substitute for the function of *E6* as an oncogene, primarily by activating cell growth-promoting signalling [23]. Therefore, E8 may play the role of E5 or E6 because it is located in a similar position in the genome (nt 1–204). Yet, BPV 04AC14 E8 shares a low degree of amino acid identity with BPV3 E8 (34.3% identity and 47.8% similarity).

The BPV 04AC14 E7 protein exhibits one conserved zinc-binding domain (ZnBD: **C**X_2_**C**X_29_**C**X_2_**C) (**Fig 2B) [24] and possesses a conserved retinoblastoma tumour-suppressor protein-binding domain (pRbBD: **L**x**C**x**E**) at amino acid positions 47–83 and 24–28 (Fig 2B) [21,25]. The pRbBD is associated with oncogenesis in human papillomaviruses 16 (HPV16) and 18 (HPV18) [26,27]. The major cellular target for PV E7 and other viral oncoproteins involved in cell transformation is the retinoblastoma tumour suppressor (pRb) [28,29]. The ZnBD in E7, together with pRbBD, are responsible for the immortalization and transformation of host cells [30]. All *Xipapillomavirus* members contain a pRbBD, which suggests that it could play a significant biological role for epitheliotropic papillomaviruses [31,32].

The putative E1 protein (with helicase function) has an ATP-dependent helicase **G**X_4_**GK**[**T/S**] (**G**PPNT**GKS** in BPV 04AC14) domain at amino acid positions 441–448 [33]. The novel PV E1 has a cyclin A interaction motif (RXL), which is thought to be important for the initiation of papillomavirus replication [34].

The E1 and E2 proteins are essential for genome transcription and replication [35]. The 04AC14 E2 protein harbours a hinge region with a sequence rich in arginine, serine, and glycine residues. The protein has 73.6% amino acid identity with BPV3 E2. One leucine–zipper domain (**L**_X_6**L**X_6_**L**X_6_**L**) was identified at E2 (aa 331–351) [36]. The BPV 04AC14 E4 ORF is contained within the E2 gene and is 63.9% similar to BPV3 E4. This composition is similar in *Xipapillomavirus* (BPV4, 10, 11, 12), *Epsilonpapillomavirus* (BPV8) and *Dyoxipapillomavirus* (BPV7) [21,32,37–40].

The late regions, *L1* and *L2*, were predicted to encode the major and minor capsid proteins, respectively. Both capsid proteins contain a nuclear localization sequence (NLS), which consists of a high proportion of positively charged residues (K and R) in their C-terminal ends that are likely to play a role in the nuclear translocation of L1 and L2 during the viral life cycle. Furthermore, the L1 gene has been chosen as yardstick for building PV comparisons, and taxonomic categories are based on the percentages of identity at the nucleotide level in this gene [5].

A new PV type can be proposed if the *L1* gene sequence shares less than 90% identity with the closest known PV type [5,7,41]. Here, it can be observed that the BPV 04AC14 *L1* gene diverges 21% in relation to the nucleotide sequence of the closest BPV type (BPV3). Based on this criterion, the PV strain identified in this study should be designated as a novel PV type. Different PV genera share less than 60% nucleotide sequence identity in the L1 ORF. Our results indicate that 04AC14 BPV should be regarded as a new PV type within the genus *Xipapillomavirus*.

**Fig 3:**
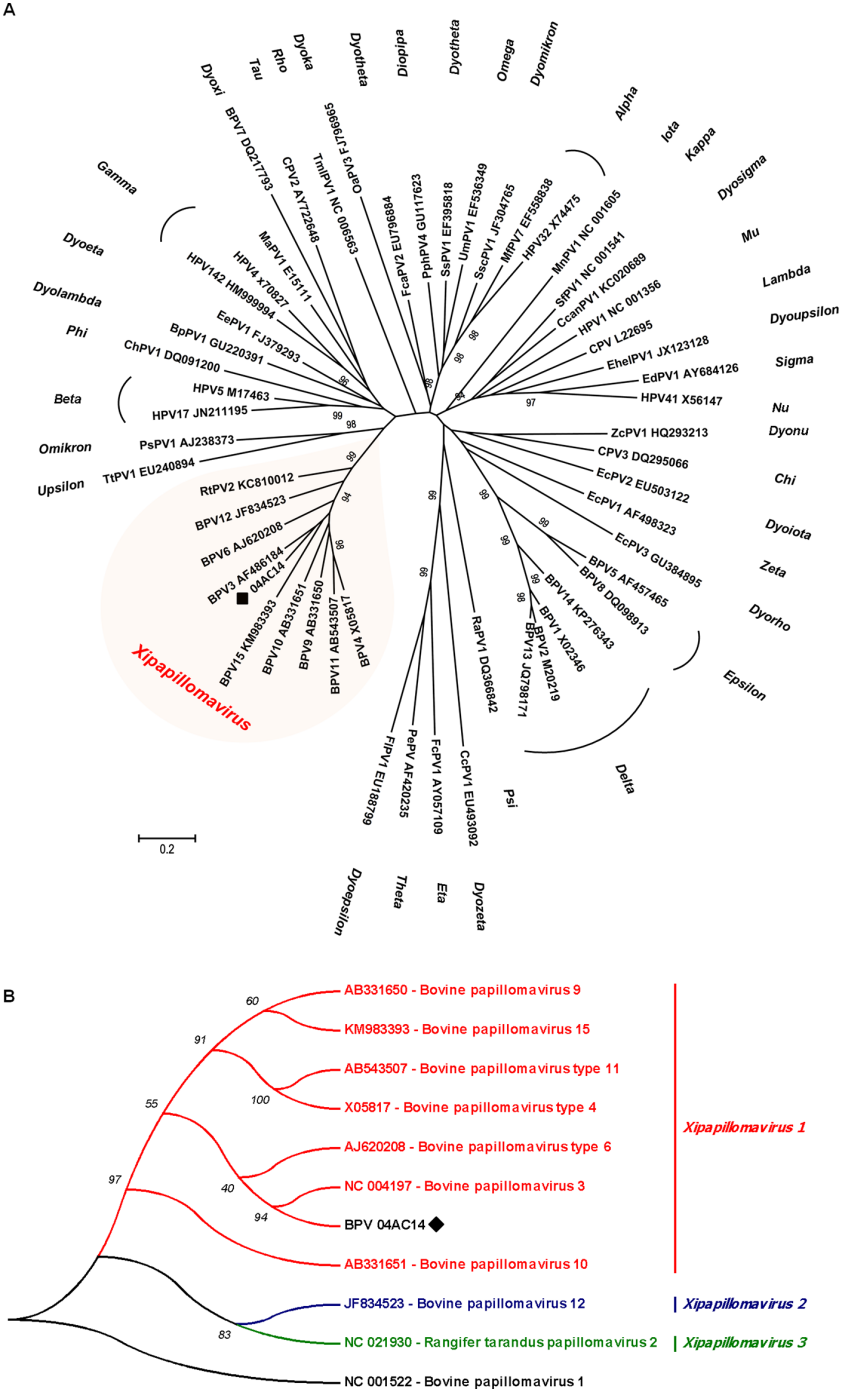
Maximum likelihood phylogenetic trees. **(A)** Maximum likelihood phylogenetic tree of *L1* gene sequences of genus-representative papillomaviruses. Nucleotide sequences were compared to those of 54 papillomavirus sequences, representing all known papillomavirus genera. The sequences were retrieved from GenBank. Bootstrap values are indicated above the branches. The putative new BPV type introduced here is indicated with a black diamond. (B) Maximum likelihood phylogenetic tree of the *Xipapillomavirus* genus. Nucleotide sequences of the *L1* gene were compared to those of nine bovine *Xipapillomavirus* sequences, representing the three known *Xipapillomavirus* species. The sequences were retrieved from GenBank. Bootstrap values are indicated above the branches. The putative new BPV type is indicated with a black diamond.

Additionally, a phylogenetic tree was reconstructed with optimized alignments based on the nucleotide sequence of the *L1* gene. Using a set of genus-representative sequences of PV, BPV 04AC14 clustered in the *Xipapillomavirus* genus along with most known bovine PVs (Fig 3A).

According to the new ICTV *Papillomavirus* taxonomy proposal, the *Xipapillomavirus* genus comprises three papillomavirus species: *Xipapillomavirus 1* (BPV3, 4, 6, 9, 10, 11 and BPV15), *Xipapillomavirus 2* (BPV12) and *Xipapillomavirus 3* (*Rangifer tarandus* papillomavirus 2). A phylogenetic tree reconstructed with all members of the *Xipapillomavirus* genus and BPV 04AC14 is shown in Fig 3B. BPV 04AC14 grouped in the *Xipapillomavirus 1* branch, which also contains BPV3, 4, 6, 9, 10, 11 and 15 (Fig 3B). This result, combined with the genomic and L1 gene identities of BPV3 (the prototype of *Xipapillomavirus 1* species), clearly demonstrate that BPV 04AC14 is a *Xipapillomavirus 1* species. The phylogenetic evidence from the present study indicates that BPV 04AC14 is closely related to bovine epitheliotropic papillomaviruses.

### Histopathological findings

**Fig 4:**
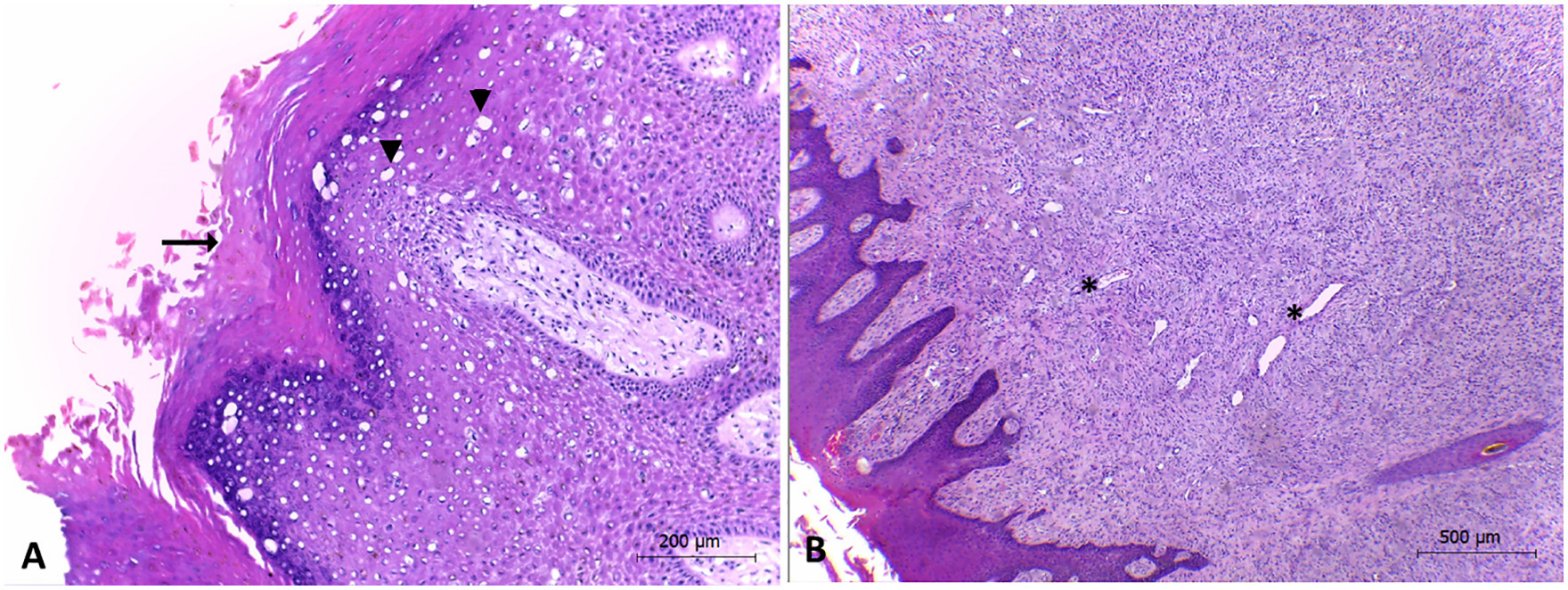
Exophytic papillomatous proliferation of the epithelium. (A) Exophytic papillomatous proliferation, acanthosis and multifocal spongiosis (arrowhead) and multifocal ortokeratosis (arrow) (obj. 10X). (B) The dermis shows fibroplasia and moderate neovascularization (*) (obj. 4X).

Based on the histopathological findings, the diagnosis of epidermal papillomatosis was confirmed for specimen 04AC14. Neoplastic tissue of sample 04AC14 consisted of exophytic papillomatous lesions, epithelium proliferation, and well-differentiated cells, showing marked acanthosis (Fig 4A and 4B). This pattern has also been observed in other BPV-associated lesions [21,42,43]. *Xipapillomaviruses* infect only epithelial cells to induce true epithelial papillomas [3,32,38]. Some *Xipapillomavirus* types have been reported to cause teat and udder papillomatosis in dairy cattle worldwide [42–44]. Such lesions inflict serious economic losses on the dairy industry. The identification of distinct types/species of BPVs may be highly important not only to improve knowledge on BPV biology but also to aid in defining the appropriate antigens for candidate vaccines.

## Conclusion

A new putative BPV type—BPV 04AC14 –is introduced. The *L1* gene shares ≤90% identity with previously described BPVs (Table 1). The reconstructed phylogenetic tree with members of the *Xipapillomavirus* genus reveals that BPV 04AC14 is clearly a new member of this genus (Fig 3A). The genome of BPV 04AC14 aligned close to the *Xipapillomavirus 1* branch, which also contains BPV3, the representative species of the genus (Fig 3B). These findings add to the expanding genetic diversity of bovine papillomaviruses. Additionally, because there was no evidence of BPV co-infection in the sample, we can infer that BPV 04AC14 is implicated in the aetiology of bovine papillomatosis.

## Supporting Information

S1 File (DOCX)

## References

[1] Rector A, Van Ranst M (2013) Animal papillomaviruses. Virology 445: 213–223. 10.1016/j.virol.2013.05.007 23711385

[2] Bravo IG, de Sanjose S, Gottschling M (2010) The clinical importance of understanding the evolution of papillomaviruses. Trends Microbiol 18: 432–438. 10.1016/j.tim.2010.07.008 20739182

[3] Borzacchiello G, Roperto F (2008) Bovine papillomaviruses, papillomas and cancer in cattle. Vet Res 39: 45 10.1051/vetres:2008022 18479666

[4] Campo MS (2002) Animal models of papillomavirus pathogenesis. Virus Res 89: 249–261. 12445664 10.1016/s0168-1702(02)00193-4

[5] Bernard HU, Burk RD, Chen Z, van Doorslaer K, zur Hausen H, et al (2010) Classification of papillomaviruses (PVs) based on 189 PV types and proposal of taxonomic amendments. Virology 401: 70–79. 10.1016/j.virol.2010.02.002 20206957 PMC3400342

[6] Van Doorslaer K, Tan Q, Xirasagar S, Bandaru S, Gopalan V, et al (2013) The Papillomavirus Episteme: a central resource for papillomavirus sequence data and analysis. Nucleic Acids Res 41: D571–578. 10.1093/nar/gks984 23093593 PMC3531071

[7] de Villiers EM, Fauquet C, Broker TR, Bernard HU, zur Hausen H (2004) Classification of papillomaviruses. Virology 324: 17–27. 15183049 10.1016/j.virol.2004.03.033

[8] Rector A, Tachezy R, Van Ranst M (2004) A sequence-independent strategy for detection and cloning of circular DNA virus genomes by using multiply primed rolling-circle amplification. J Virol 78: 4993–4998. 15113879 10.1128/JVI.78.10.4993-4998.2004 PMC400362

[9] Niel C, Diniz-Mendes L, Devalle S (2005) Rolling-circle amplification of Torque teno virus (TTV) complete genomes from human and swine sera and identification of a novel swine TTV genogroup. J Gen Virol 86: 1343–1347. 15831945 10.1099/vir.0.80794-0

[10] Dezen D, Rijsewijk FA, Teixeira TF, Holz CL, Cibulski SP, et al (2010) Multiply-primed rolling-circle amplification (MPRCA) of PCV2 genomes: applications on detection, sequencing and virus isolation. Res Vet Sci 88: 436–440. 10.1016/j.rvsc.2009.10.006 19917510

[11] Rector A, Tachezy R, Van Doorslaer K, MacNamara T, Burk RD, et al (2005) Isolation and cloning of a papillomavirus from a North American porcupine by using multiply primed rolling-circle amplification: the Erethizon dorsatum papillomavirus type 1. Virology 331: 449–456. 15629787 10.1016/j.virol.2004.10.033

[12] Forslund O, Antonsson A, Nordin P, Stenquist B, Hansson BG (1999) A broad range of human papillomavirus types detected with a general PCR method suitable for analysis of cutaneous tumours and normal skin. J Gen Virol 80 (Pt 9): 2437–2443. 10501499 10.1099/0022-1317-80-9-2437

[13] Rijsewijk FA, Dos Santos HF, Teixeira TF, Cibulski SP, Varela AP, et al (2011) Discovery of a genome of a distant relative of chicken anemia virus reveals a new member of the genus Gyrovirus. Arch Virol 156: 1097–1100. 10.1007/s00705-011-0971-6 21442232

[14] Bankevich A, Nurk S, Antipov D, Gurevich AA, Dvorkin M, et al (2012) SPAdes: a new genome assembly algorithm and its applications to single-cell sequencing. J Comput Biol 19: 455–477. 10.1089/cmb.2012.0021 22506599 PMC3342519

[15] Ye J, McGinnis S, Madden TL (2006) BLAST: improvements for better sequence analysis. Nucleic Acids Res 34: W6–9. 16845079 10.1093/nar/gkl164 PMC1538791

[16] Edgar RC (2004) MUSCLE: multiple sequence alignment with high accuracy and high throughput. Nucleic Acids Res 32: 1792–1797. 15034147 10.1093/nar/gkh340 PMC390337

[17] Posada D, Crandall KA (2001) Selecting models of nucleotide substitution: an application to human immunodeficiency virus 1 (HIV-1). Mol Biol Evol 18: 897–906. 11371577 10.1093/oxfordjournals.molbev.a003890

[18] Tamura K, Peterson D, Peterson N, Stecher G, Nei M, et al (2011) MEGA5: molecular evolutionary genetics analysis using maximum likelihood, evolutionary distance, and maximum parsimony methods. Mol Biol Evol 28: 2731–2739. 10.1093/molbev/msr121 21546353 PMC3203626

[19] Zheng ZM, Baker CC (2006) Papillomavirus genome structure, expression, and post-transcriptional regulation. Front Biosci 11: 2286–2302. 16720315 10.2741/1971 PMC1472295

[20] Desaintes C, Demeret C (1996) Control of papillomavirus DNA replication and transcription. Semin Cancer Biol 7: 339–347. 9284526 10.1006/scbi.1996.0043

[21] Zhu W, Dong J, Shimizu E, Hatama S, Kadota K, et al (2012) Characterization of novel bovine papillomavirus type 12 (BPV-12) causing epithelial papilloma. Arch Virol 157: 85–91. 10.1007/s00705-011-1140-7 22033594

[22] Bravo IG, Felez-Sanchez M (2015) Papillomaviruses: Viral evolution, cancer and evolutionary medicine. Evol Med Public Health 2015: 32–51. 10.1093/emph/eov003 25634317 PMC4356112

[23] Jackson ME, Pennie WD, McCaffery RE, Smith KT, Grindlay GJ, et al (1991) The B subgroup bovine papillomaviruses lack an identifiable E6 open reading frame. Mol Carcinog 4: 382–387. 1654923 10.1002/mc.2940040510

[24] Lehoux M, D'Abramo CM, Archambault J (2009) Molecular mechanisms of human papillomavirus-induced carcinogenesis. Public Health Genomics 12: 268–280. 10.1159/000214918 19684440 PMC4654617

[25] Dahiya A, Gavin MR, Luo RX, Dean DC (2000) Role of the LXCXE binding site in Rb function. Mol Cell Biol 20: 6799–6805. 10958676 10.1128/mcb.20.18.6799-6805.2000 PMC86207

[26] Scase T, Brandt S, Kainzbauer C, Sykora S, Bijmholt S, et al (2010) Equus caballus papillomavirus-2 (EcPV-2): an infectious cause for equine genital cancer? Equine Vet J 42: 738–745. 10.1111/j.2042-3306.2010.00311.x 21039805

[27] Munday JS, Kiupel M (2010) Papillomavirus-associated cutaneous neoplasia in mammals. Vet Pathol 47: 254–264. 10.1177/0300985809358604 20106770

[28] Stevaux O, Dyson NJ (2002) A revised picture of the E2F transcriptional network and RB function. Curr Opin Cell Biol 14: 684–691. 12473340 10.1016/s0955-0674(02)00388-5

[29] Harbour JW, Dean DC (2000) Chromatin remodeling and Rb activity. Curr Opin Cell Biol 12: 685–689. 11063932 10.1016/s0955-0674(00)00152-6

[30] Liu X, Clements A, Zhao K, Marmorstein R (2006) Structure of the human Papillomavirus E7 oncoprotein and its mechanism for inactivation of the retinoblastoma tumor suppressor. J Biol Chem 281: 578–586. 16249186 10.1074/jbc.M508455200

[31] Chan HM, Smith L, La Thangue NB (2001) Role of LXCXE motif-dependent interactions in the activity of the retinoblastoma protein. Oncogene 20: 6152–6163. 11593423 10.1038/sj.onc.1204793

[32] Hatama S, Ishihara R, Ueda Y, Kanno T, Uchida I (2011) Detection of a novel bovine papillomavirus type 11 (BPV-11) using xipapillomavirus consensus polymerase chain reaction primers. Arch Virol 156: 1281–1285. 10.1007/s00705-011-0970-7 21424729

[33] Liu X, Schuck S, Stenlund A (2010) Structure-based mutational analysis of the bovine papillomavirus E1 helicase domain identifies residues involved in the nonspecific DNA binding activity required for double trimer formation. J Virol 84: 4264–4276. 10.1128/JVI.02214-09 20147403 PMC2863750

[34] Ma T, Zou N, Lin BY, Chow LT, Harper JW (1999) Interaction between cyclin-dependent kinases and human papillomavirus replication-initiation protein E1 is required for efficient viral replication. Proc Natl Acad Sci U S A 96: 382–387. 9892642 10.1073/pnas.96.2.382 PMC15145

[35] Lu JZ, Sun YN, Rose RC, Bonnez W, McCance DJ (1993) Two E2 binding sites (E2BS) alone or one E2BS plus an A/T-rich region are minimal requirements for the replication of the human papillomavirus type 11 origin. J Virol 67: 7131–7139. 8230435 10.1128/jvi.67.12.7131-7139.1993 PMC238175

[36] Van Doorslaer K, Rector A, Jenson AB, Sundberg JP, Van Ranst M, et al (2007) Complete genomic characterization of a murine papillomavirus isolated from papillomatous lesions of a European harvest mouse (Micromys minutus). J Gen Virol 88: 1484–1488. 17412977 10.1099/vir.0.82615-0

[37] Patel KR, Smith KT, Campo MS (1987) The nucleotide sequence and genome organization of bovine papillomavirus type 4. J Gen Virol 68 (Pt 8): 2117–2128. 3039043 10.1099/0022-1317-68-8-2117

[38] Hatama S, Nobumoto K, Kanno T (2008) Genomic and phylogenetic analysis of two novel bovine papillomaviruses, BPV-9 and BPV-10. J Gen Virol 89: 158–163. 18089739 10.1099/vir.0.83334-0

[39] Tomita Y, Literak I, Ogawa T, Jin Z, Shirasawa H (2007) Complete genomes and phylogenetic positions of bovine papillomavirus type 8 and a variant type from a European bison. Virus Genes 35: 243–249. 17265141 10.1007/s11262-006-0055-y

[40] Ogawa T, Tomita Y, Okada M, Shirasawa H (2007) Complete genome and phylogenetic position of bovine papillomavirus type 7. J Gen Virol 88: 1934–1938. 17554025 10.1099/vir.0.82794-0

[41] de Villiers EM (2013) Cross-roads in the classification of papillomaviruses. Virology 445: 2–10. 10.1016/j.virol.2013.04.023 23683837

[42] Maeda Y, Shibahara T, Wada Y, Kadota K, Kanno T, et al (2007) An outbreak of teat papillomatosis in cattle caused by bovine papilloma virus (BPV) type 6 and unclassified BPVs. Vet Microbiol 121: 242–248. 17239550 10.1016/j.vetmic.2006.12.015

[43] Batista MV, Silva MA, Pontes NE, Reis MC, Corteggio A, et al (2013) Molecular epidemiology of bovine papillomatosis and the identification of a putative new virus type in Brazilian cattle. Vet J 197: 368–373. 10.1016/j.tvjl.2013.01.019 23489845

[44] Tozato CC, Lunardi M, Alfieri AF, Otonel RA, Di Santis GW, et al (2013) Teat papillomatosis associated with bovine papillomavirus types 6, 7, 9, and 10 in dairy cattle from Brazil. Braz J Microbiol 44: 905–909. 10.1590/S1517-83822013005000057 24516429 PMC3910210

