# Supplementary material for: Novel Bovine Papillomavirus Type Discovered by Rolling-Circle Amplification Coupled with Next-Generation Sequencing
Source: PLoS One. 2016 Sep 8;11(9):e0162345. doi: 10.1371/journal.pone.0162345 (PMC5015974; doi:10.1371/journal.pone.0162345)
Supplement: S1 File — (DOCX) [file pone.0162345.s001.docx]

**Supplemental file S1**

**Novel Bovine Papillomavirus Type Discovered by Rolling-Circle Amplification Coupled with Next-Generation Sequencing**

**DNA extraction, PV consensus PCR and Sanger sequencing**

For DNA isolation, ~1 g of papillomatous tissue of specimen 04AC14 was processed as previously described [[1](#_ENREF_1)]. DNA extraction was carried out using a silica-based protocol [[2](#_ENREF_2)]. Purified DNA was eluted in TE buffer and stored at −20°C. The DNA quality and quantity were evaluated by spectrophotometry and fluorometry, respectively.

Partial amplification of the L1 gene was performed with FAP59 and FAP64 primers [[3](#_ENREF_3)]. Briefly, 100 ng of extracted DNA was mixed with 20 pmol of each primer, 1.5 mM of MgCl_2_, 200 μM of dNTPs, 1 U of Platinum Taq DNA polymerase and 1X PCR buffer (all reagents from Invitrogen, USA). The cycling conditions and reaction analysis were as previously described [[1](#_ENREF_1)]. Sanger sequencing followed by BLASTn analysis was used to identify the PV aetiology [[1](#_ENREF_1)].

**Supplemental references**

1. da Silva FR, Daudt C, Streck AF, Weber MN, Filho RV, et al. (2015) Genetic characterization of Amazonian bovine papillomavirus reveals the existence of four new putative types. Virus Genes 51: 77-84.

2. Boom R, Sol CJ, Salimans MM, Jansen CL, Wertheim-van Dillen PM, et al. (1990) Rapid and simple method for purification of nucleic acids. J Clin Microbiol 28: 495-503.

3. Forslund O, Antonsson A, Nordin P, Stenquist B, Hansson BG (1999) A broad range of human papillomavirus types detected with a general PCR method suitable for analysis of cutaneous tumours and normal skin. J Gen Virol 80 ( Pt 9): 2437-2443.
